# Intravenous Amide Anesthetics to Treat Pain Associated with Renal Colic in the Emergency Department: a Systematic Review

**Published:** 2020-03-18

**Authors:** Andrew C. C. Miller, Colton Faza, Alberto A Castro Bigalli, Abbas M. Khan, Kerry A. Sewell, Alexandra King, Amir Vahedian-Azimi, Shahriar Zehtabchi

**Affiliations:** 1 Department of Emergency Medicine, Vidant Medical Center, East Carolina University Brody School of Medicine, Greenville, NC, USA; 2 The Morzak Collaborative, Greenville, NC, USA; 3 East Carolina University Brody School of Medicine, Greenville, NC, USA; 4 William E. Laupus Health Sciences Library, East Carolina University, Greenville, NC, USA; 5 Emergency Medicine and Toxicology Department, Vidant Medical Center, Greenville, NC, USA; 6 Trauma Research Center, Nursing Faculty, Baqiyatallah University of Medical Sciences, Tehran, Iran; 7 Department of Emergency Medicine, State University of New York (SUNY) Downstate Medical Center, Brooklyn, NY, USA

**Keywords:** Renal Colic, Kidney Calculi, Lidocaine, Analgesia, Emergency Service, Hospital

## Abstract

**Introduction:**

Renal colic affects 12% of the U.S. population, accounting for nearly 1% of emergency department (ED) visits. Current recommendations advocate narcotic-limiting multimodal analgesia regimens. The objective of this review is to determine if in patients with renal colic (Population), intravenous (IV) amide anesthetics (Intervention) result in better pain control, lower requirements for rescue analgesia, or less adverse medication effects (outcome) compared to placebo, non-steroidal anti-inflammatory drugs (NSAIDs), or opiates (Comparisons).

**Methods:**

Scholarly databases and relevant bibliographies were searched using a pre-designed systematic review protocol and registered with PROSPERO. Inclusion criteria were: (1) randomized clinical trial (RCT), (2) age ≥ 18 years, (3) confirmed or presumed renal colic, (4) amide anesthetic administered IV. Eligible comparison groups included: placebo, conventional therapy, acetaminophen, NSAID, or opiate. The primary outcome was pain intensity at baseline, 30, 60, and 120 minutes. Trial quality was graded, and risk-of-bias was assessed.

**Results:**

Of the 3930 identified references, 4 RCTs (479 participants) were included. One trial (n=240) reported improved analgesia with IV lidocaine (Lido_IV_) plus metoclopramide, compared to morphine. All other trials reported unchanged or less analgesia compared to placebo, ketorolac, or fentanyl. Very severe heterogeneity (I^2^= 88%) precluded pooling data.

**Conclusion:**

Current evidence precludes drawing a firm conclusion on the efficacy or superiority of Lido_IV_ over traditional therapies for ED patients with renal colic. Evidence suggests Lido_IV_ may be an effective non-opiate analgesic alliterative; however, it’s efficacy may not exceed that of NSAIDs or opiates. Further study is needed to validate the potential improved efficacy of Lido_IV_ plus metoclopramide.

## Introduction

Pain is the most common reason for emergency department (ED) visits in the United States (U.S.) (1), and its management requires mastery of multimodal approaches to achieve safe and effective analgesia. Nephrolithiasis and renal colic affect approximately 12% of the U.S. population (up to 5% in China) (2) and accounts for nearly 1% of ED visits and hospital admissions in the U.S. (2–5). For patients with prior stones, 10-year recurrence rates approach 50% (3,6). The pain of renal colic origin is multifactorial and is related to obstruction of urinary flow with subsequent increase in prostaglandin-mediate ureteral spasm (6,7). 

As most renal calculi pass spontaneously, acute management should focus on rapid analgesia, diagnosis confirmation, and recognition of complications requiring immediate intervention (7). Approximately 85% of ED patients with renal colic are treated with analgesics (3). Whether used alone or in combination, NSAIDs and opioids constitute the primary therapeutic medications in ED management of renal colic (8). Each drug class possesses potentially unfavorable side effects and contraindications. Disadvantages to NSAIDs include lack of titratability, nausea, epigastric pain, and contraindications including renal insufficiency, peptic ulcer disease, the elderly (age >70 years), and concomitant use of anticoagulation or antiplatelet agents (9). Opioid administration in turn may lead to nausea, vomiting, pruritus, lethargy, bradycardia, hypotension, or respiratory depression, and may be relatively contraindicated for patients with a history of opioid abuse or dependence. Despite efficacy and possibly favorable side-effect profile of NSAIDS over opiates (6), 43% of patients are treated with an opiate, and 70% are prescribed an opiate on discharge (3). Moreover, ED opioid administration and prescription has been linked to an increased risk of recurrent opioid use (10). Current practice is moving towards recent U.S. Food and Drug Administration (FDA) goals that emphasize analgesia through multimodal regimens that decrease opiate use (11). Insufficient data regarding the efficacy of alternative regimens and their side-effect profiles have hampered efforts to move away from opiate heavy regimens (12). There is a need for identification and validation of safe and effective analgesia techniques for renal colic, which limit narcotic consumption and prescription, and provide treatment alternatives for patients who are unable to tolerate or have serious contraindications to NSAIDs or opiates (12). 

Intravenous local anesthetic use has emerged as an opiate-sparing alternative in treatment of renal colic. Local anesthetics halt impulse initiation and transmission processes in neuronal axons, and may be categorized into two major chemical classes: amino esters and amino amides (6). Amide local anesthetics are widely used for topical and local anesthesia, and as systemic antiarrhythmics (1). Lidocaine, an amino amide, has been described to have analgesic, anti-hyperalgesic, anti-inflammatory, and anti-bacterial properties (1,13,14). The analgesic effect from systemic administration affects both the peripheral and central nervous system (15). Lidocaine decreases excitability and conduction of unmyelinated C fibers, and intravenous lidocaine (Lido_IV_) suppresses post-synaptic reflexes in the spinal dorsal horn (15). Its mechanisms include reversible inhibition of voltage-gated open and inactivated sodium channels and G-protein-coupled receptors (3,13,15,16). Central anti-nociceptive effects are mediated through actions on muscarinic and nicotinic receptors, which in turn increase intraspinal acetylcholine release to reinforce the inhibitory descending pain pathway (15). Anti-hyperalgesic effects are mediated through the N-methyl-D-aspartate (NMDA) receptor (14,15). 

Lido_IV_ has a desirable pharmacokinetic profile, with a rapid onset and long duration-of-action (half-life 60-120 min), but the analgesic effects may last longer (1,17). Approximately, 90% of lidocaine is metabolized in the liver by dealkylation to lower potency active metabolites monoethylglycinexylidide (MEGX) and glycinexylidide (GX) (13,15), while ≤10% is excreted unchanged in the urine (15).

Lido_IV_ application for ED patients with renal colic has been reported to improve pain intensity, time to pain relief, and nausea in randomized clinical studies (8,18–21), non-randomized clinical studies (22), and case series (17,23–25), and may be considered as a viable non-opioid addition or an alternative to traditional treatment modalities. We investigated the evidence on using intravenous amide anesthetics for analgesia and opioid sparing effects in patients with acute renal colic. The objective of this project is to address the following research question: In patients with renal colic (Population) do intravenous amide anesthetics such as lidocaine (Intervention) improve pain intensity, need for rescue analgesia, opiate consumption, or adverse events (Outcomes) compared to placebo, NSAIDs, or opiates (Comparisons)? 

## Methods

This systematic review followed the steps outlined in Preferred Reporting Items for Systematic Reviews and Meta-Analyses (PRISMA) (26). A systematic protocol was developed *a priori* and registered with PROSPERO (# CRD42019130355). 

The primary outcome was pain intensity at baseline and 15, 30, 60, and 120 minutes post-treatment. The secondary outcomes were: (1) need for rescue analgesia at 30 or 60 minutes, (2) time to pain free, (3) treatment failure, and (4) adverse events. 

A librarian-performed systematic search strategy was conducted (Supplemental Digital Content 1) in Cochrane CENTRAL, CINAHL, Embase, Latin American and Caribbean Health Sciences Literature (LILACS), Medline, Scopus, and Web of Science (WoS). Additional investigator-performed structured searches were conducted in: China National Knowledge Infrastructure (CHKD-CNKI), information/Chinese Scientific Journals database (CSJD-VIP), Directory of Open Access Journals (DOAJ), IEEE-Xplorer, Magiran, Scientific Information Database (SID), TÜBİTAK ULAKBİM, Russian Science Citation Index (RSCI), Korean Journal Database (KCI), and Scientific Electronic Library Online (SciELO). Relevant bibliographies were searched. Searches were not limited by date, language, or publication status. Clinical trial registries were searched to limit publication bias, including: ClinicalTrials.gov, World Health Organization International Clinical Trials Registry Platform (WHO ICTRP), and the Australian New Zealand Clinical Trials Registry (ANZCTR). Abstracts of the conference proceedings of the relevant disciplines (emergency medicine, urology, nephrology, pain management) were searched (past 5 years). When the presented data were incomplete, the authors were contacted to obtain the missing information. These trials were only included if the authors responded to correspondence affirmatively with the requested information. 

Inclusion criteria were: (1) randomized controlled human clinical trial, (2) patients aged ≥ 18 years, (3) presumed or confirmed renal colic, (4) amino amide anesthetic administered intravenously (eg. Lido_IV_) compared to placebo or another analgesic. Data of pain intensity that measured as either a 10 cm visual analogue scale (VAS) or 10-point numeric rating scale (NRS) were summarized. Significant improvement in pain intensity was defined as improvement in ≥ 3 cm or points on VAS or NRS, respectively. Rescue analgesia was defined as any analgesia medication administered following the study drug. 

Exclusion criteria were: (1) non-randomized study design, (2) studies enrolling patients aged < 18 years, (3) drug administration by routes other than intravenous, (4) studies published only in abstract form (or unpublished) for which the authors did not respond to correspondence by providing the requested information.

Reference management and application of inclusion/exclusion criteria was performed using Covidence (Covidence, Melbourne, Australia). Four authors reviewed the titles and abstracts to determine inclusion eligibility. Four authors extracted study data. Any disagreements were resolved by consensus. 

Four authors independently assessed the risk-of-bias (RoB) using two validated tools: (1) Grading of Recommendations, Assessment, Development and Evaluations (GRADE) (27), and RoB 2.0: "Revised tool for Risk of Bias in randomized trials” (28). The authors considered methods of randomization and allocation, blinding (of treatment administrator, participants, and outcome assessors), selective outcome reporting (e.g. failure to report adverse events), incomplete outcome data, and sample size calculation. Each trial was graded as high, low or unclear risk of bias (RoB) for each criterion. Publication bias was assessed using both the Egger (29) and Begg-Mazumdar methods (30). Heterogeneity was evaluated using I² statistic. The confidence interval for I² was constructed using the iterative non-central chi-square distribution method of Hedges and Piggott (31). The threshold value for severe heterogenity was specified to be I² ≥50%, and very serious heterogenity was specified as I² >75%. Data pooling and meta-analysis was planned if I² <50%. 

## Results

**Figure 1:**
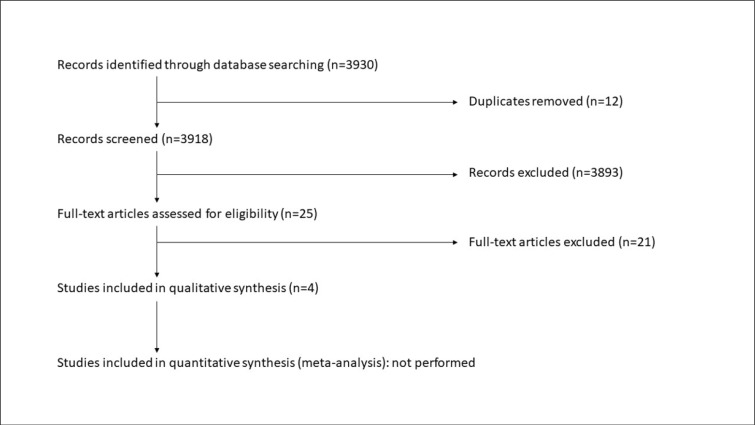
PRISMA Flow Diagram

The complete search was performed on December 19, 2018. The search strategy identified 3930 references, of which 4 RCTs (479 participants) met the inclusion criteria (8,18–20). Two ongoing studies were identified in clinical trial registries (32,33). See Figure 1 for the PRISMA flow diagram. The included studies are summarized in Table 1. One study took place in a high-income economy (USA) (8). Three were in a middle-income economy (Iran) (18–20). No studies were identified in low-income economies. Two published abstracts were excluded. The first was an RCT published only in abstract form for which the authors did not respond to correspondence (21). The second duplicated information available in a published manuscript (22). The primary reason for exclusion of full-text manuscripts was non-randomized study design. Two unpublished ongoing trials were identified (32,33). No additional studies were identified through bibliographic and conference abstracts analyses that were not previously identified through other search methods.

The GRADE assessments are presented in Table 2. RoB assessment indicated that how each study ranked regarding the risk of selection bias, performance bias, detection bias, reporting bias, and “other” bias (8,18–20). Two studies were similarly at low risk for attrition bias (8,18), whereas 2 had unclear risk of attrition bias (19,20). Lastly, 3 studies had low risk of bias due to sample size (8,19,20), whereas one had unclear RoB (18). Additionally, all of the included studies were double-blind (8,18–20). Although all 4 studies had a control arm, but only one had a placebo arm (lidocaine + morphine vs. placebo + morphine) (18). One study reported no patient attrition (8), whereas one reported 19% attrition (18), and two did not report attrition data (19,20). Furthermore, each included study reported their intended primary outcomes. Moreover, one study reported adverse events (AE) (20), 2 reported no AEs (8,18), and one did not report on AEs (19). All but one study reported the method of sample size calculation (18). 

All included studies gave adequate information regarding diagnostic criteria, namely that patients were diagnosed with presumed or confirmed renal colic (8,18–20). Heterogeneity was noted in the methods and timing of pain intensity assessments. Three studies utilized a 10 cm VAS scale (18–20), and 1 utilized a 10-point NRS (8). Pain intensity was assessed at 15 minutes in 3 studies (8,19,20), at 30 minutes in 4 studies (8,18–20), at 60 minutes in 3 studies (8,18,20), and at 120 minutes in 2 studies (8,18). Only one study assessed time to pain-free (18), one assessed treatment failure (19), and one assessed the need for rescue analgesia (at 30 and 60 minutes) (8). 

**Figure 2:**
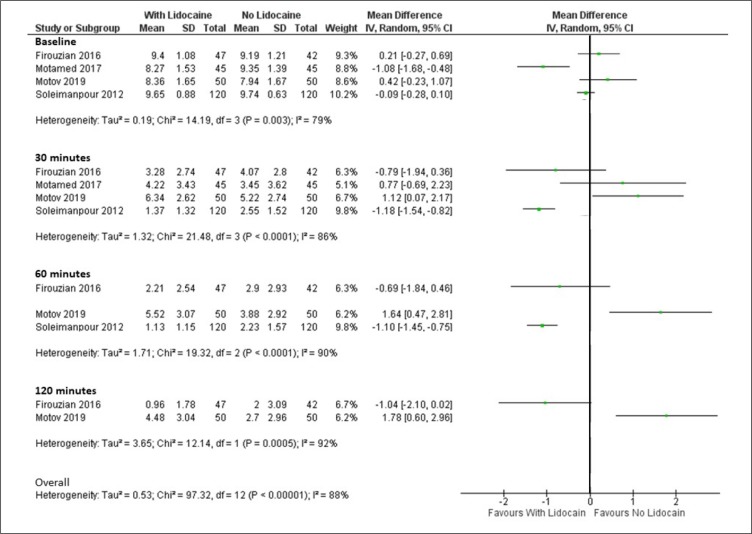
Forrest plot of pain scores in lidocaine-containing versus no lidocaine regimens

**Figure 3:**
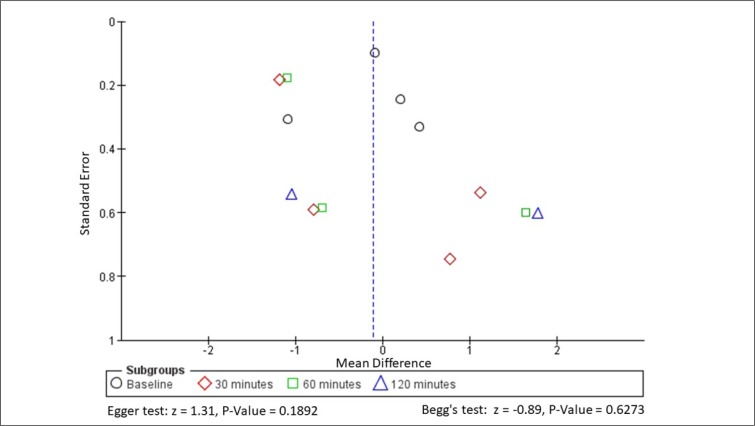
Funnell plot and publication bias assessment

All four included trials gave adequate statistical descriptions; including appropriate use of statistical tests (8,18–20). No significant baseline differences were noted between groups (8,18–20). With the exception of one trial that reported better pain control with lidocaine (compared to IV morphine) at 10, 15, and 30 minutes post-administration (20), all other trials reported similar (or worse) pain intensity compared to placebo (18), ketorolac (8), or fentanyl (Figure 3) (19). Since not all trials reported immediate (10 minutes post-administration) or longer pain relief (60 or 120 minutes post-administration), it was not possible to draw a conclusion about the immediate or long-term analgesia effects of Lido_IV_. A forest plot depicting pain score comparisons between regimens with and without lidocaine is provided in Figure 2.

Neither the Egger (z = 1.31, p-value = 0.1892) nor Begg-Mazumdar method (z = -0.89, P-Value = 0.6273) identified evidence of publication bias (Figure 3). The planned meta-analysis was not performed due to very severe heterogeneity (I^2^ >75%). 

## Discussion

This project aimed to clarify the clinical efficacy of Lido_IV_ for decreasing the pain intensity and analgesic requirements associated with acute renal colic. Although substantial improvement was not noted over comparators, overall, some observations warrant further discussion. Of note, the methodology of the Soleimanpour et al. study that *did* report improvement over morphine differed from the other studies in a few important ways, namely: (1) coadministration with metoclopramide and (2) conservative IV fluid strategy (20).

Metoclopramide is a procaine amide structural analogue that may exert both antiemetic and analgesic effects. Proposed mechanisms include calcium channel-, opiate-, and prolactin-mediated mechanisms. The latter contributes to the analgesic action of the endogenous opioid system as evidenced by its reversibility by naloxone. Additionally, metoclopramide is a dopamine antagonist, increasing acetylcholine levels at neuro-effector junctions and post-ganglionic nerve terminals by inhibiting the action of acetylcholinesterase. Metoclopramide has been described to have antispasmodic effects on ureteral smooth muscle (34). Studies have described its analgesic efficacy in acute renal colic to exceed those of the isosorbide dinitrate (35), morphatropin (36), tenoxicam (37), and xintonding (38); however, findings were not significant for metoclopramide *plus* dipyrone vs. ketorolac alone (39), or metoclopramide *plus* pethidine vs. morphine alone (40). It has been described that combining lidocaine with metoclopramide may increase analgesia over lidocaine alone (41). It remains unclear whether an analgesia augmenting synergistic relationship exists between metoclopramide and the amide anesthetics.

The second way in which the study by Soleimanpour et al. differed from the others was fluid management strategy. In many regions, it is commonplace to treat patients with acute renal colic with forced IV fluids; however, this remains controversial. Large volumes of IV fluids are often administered to produce a diuresis that mechanically "flushes out" the stone. However, the benefit of this approach has not consistently borne out in clinical practice (42,43). The Soleimanpour *et al*. protocol did not administer forced IV fluids (20), whereas the other included studies did not specify the IV fluid management strategy (8,18,19). The influence of varied hydration strategies on the outcomes of these studies remains unclear. 

In some situations, Lido_IV_ doses may need to be modified. With commonly recommended doses, lidocain’s therapeutic index remains very high and plasma concentrations stay largely below the cardiotoxic and neurotoxic threshold levels (15). For renal colic, the recommended dose of Lido_IV_ is 1.5 mg/kg (maximum 200 mg/dose) administered over 20-30 minutes (17,23,25). For patients being admitted, repeat dosing or an infusion may be used to maintain a steady-state plasma concentration: 1.5 mg/kg IV bolus, *then* 50 μg/kg/min (3.0 mg/kg) IV infusion for one hour, *then* 25 μg/kg/min (1.5 mg/kg) IV infusion for the second hour (h), *then* 12 μg/kg/min (0.7 mg/kg) IV infusion for the next 22 h, and finally 10 μg/kg/min (0.6 mg/kg) IV infusion from 24 to 48 h (15). Without a loading dose, it takes >60 min for Lido_IV_ to achieve a therapeutic steady-state plasma concentration (15). Although continuous lidocaine infusion might theoretically lead to toxicity over time, blood concentrations reported in clinical studies have remained below toxic levels ( 5 µg/ml), except for cardiac surgery trials in which higher doses were used for longer durations (15).

As hepatic blood flow appears to be a limiting factor for lidocaine metabolism (13), the reduction in hepatic blood flow in patients with congestive heart failure may prolong the elimination half-life (T_1/2_) (44). No dose adjustment is necessary in patients with moderate liver cirrhosis; however, the dose should be decreased by 50% in patients with severe cirrhosis (Child score C) (45). Additionally, first- and second-degree heart blocks could be exacerbated and progress to a higher degree block with lidocaine administration, and both cardiovascular instability and concomitant use of alpha-agonists or beta-blockers are relative contraindications (46). Moreover, lidocaine clearance is linearly altered with kidney impairment, thus the elimination T_1/2_ of lidocaine and GX (but not MEGX) is doubled in case of severe renal insufficiency (47). 

Volume of distribution is also an important factor when considering Lido_IV_ dose and metabolism. Elderly patients have an increase in apparent volume of distribution, and consequently a significantly longer elimination T_1/2_ compared to younger patients (2.7 vs. 1.6 h) (48). For elderly patients, the initial loading dose should be the same, but any continuous infusion rate should be decreased by approximately 35% (15). The increased volume of distribution similarly accounts for the prolonged clearance seen in obese patients compared to non-obese patients (48). For obese patients, the bolus or loading dose should be calculated based on the patient’s total body weight, but the continuous infusion rate should be based on the ideal body weight (15). Lastly, lidocaine crosses the placenta and the blood–brain barriers via simple passive diffusion, and is excreted in breast milk (15). Thus, the clearance rate should be taken into consideration for breastfeeding mothers to avoid toxicity in the breast-fed infant (15). 

**Table 1:** List of included studies

**Author [Year]; (Reference #)**	**Setting, design (N)**	**Intervention**	**Comparison**	**Demographics:** **Lidocaine (L)** **vs. no lidocaine (NL) **	**Primary Endpoints**	**Secondary Endpoints**
Firouzian [2016]; (18)	Iran, single center, RCT, double-blind (89)	Lidocaine 1.5 mg/kg IV *plus *morphine 0.1 mg/kg infusion over 2-4 minutes.	Morphine 0.1 mg/kg *plus *N.S. bolus infusion over 2-4 minutes.	*Age, years*: (L) 37.91±10.76, (NL) 37.95±12.6 *Sex, male*: (L) 77%, (NL) 83%	Pain intensity measured by VAS (0-10) at baseline, 5, 10, 30, 60, and 120 minutes.	(1) Time to pain free (2) Nausea intensity (3) Time to nausea free
Motamed [2017]; (19)	Iran, single center, RCT, double-blind (90)	Lidocaine 1.5 mg/kg IV infusion over 2 minutes.	Fentanyl 1.5 mcg/kg IV infusion over 2 minutes.	*Age, years*: (L) 39.08±6.64, (NL) 34.08±8.87 *Sex, male*: (L) 86.7%, (NL) 93.3%	Pain intensity measured by VAS (0-10) at baseline, 5, 10, 15, and 30 minutes.	Rescue medication at 15- & 30-minutes post-administration.
Motov [2019]; (8)	USA, single center, RCT, double-blind (150)	Lidocaine 1.5 mg/kg IV infusion over 10 minutes.	(1) Ketorolac 30 mg IV push with 10 min N.S. infusion. (2) Ketorolac 30 mg IV push *plus* Lidocaine 1.5 mg/kg IV infusion over 10 minutes.	*Age, years*: (L only) 39.34±10.95 (Combination) 42.92±10.36 (NL) 42.34±10.47 *Sex, male*: (L only) 54%, (Combination) 56%, (NL) 56%	Pain intensity measured by numerical rating scale (0-10) at baseline, 5, 10, 30, and 60 minutes.	(1) Adverse effects (2) Use of diagnostic imaging
Soleimanpour [2012]; (20)	Iran, single center, RCT, double-blind, (150)	Lidocaine 1.5 mg/kg IV slow push from 10 cc syringe.	Morphine 0.1 mg/kg IV slow push from 10 cc syringe.	*Age, years*: (L) 37.71±11.08, (NL) 35.23±12.37 *Sex, male*: (L) 28%, (NL) 72%	Pain intensity measured by VAS (0-10) at baseline, 5, 10, 15, and 30 minutes.	(1) Pain resolution measured as VAS < 3/10 for 30 minutes. (2) Rescue medication at 30 minutes. (3) Adverse effects

Abbreviations: ED means emergency department; IV means intravenous; N.S. means normal saline; RCT means randomized controlled trial; VAS means visual analogue scale.

**Table 2:** GRADE quality of evidence ratings

	**Certainty assessment**							**Certainty**
**Variable**	**№ of studies**	**Study design**	**Risk of bias**	**Inconsistency**	**Indirectness**	**Imprecision**	**Other considerations**	
Pain intensity	4	RCT	Not serious	Serious	Not serious	Not serious	None	⨁⨁⨁◯ MODERATE
Rescue medication	1	RCT	Not serious	Not Serious	Not Serious	Not Serious	Publication bias strongly suspected	⨁⨁⨁◯ MODERATE
Time to pain free	1	RCT	Not serious	Not Serious	Not Serious	Not Serious	Publication bias strongly suspected	⨁⨁⨁◯ MODERATE
Treatment failure	1	RCT	Not serious	Not Serious	Not Serious	Not Serious	Publication bias strongly suspected	⨁⨁⨁◯ MODERATE

**RCT means randomized controlled trial.**

## Limitations

Since the data from some studies was unavailable, some risk of publication and selective reporting bias exists. Additionally, all included studies were conducted in Asia and North America. It is possible that genetic differences in drug metabolism or response may account for some of the observed variation in response. We identified no other systematic reviews or meta-analyses on this topic for comparison.

## Conclusion

While we cannot draw a firm conclusion on the efficacy or superiority of intravenous lidocaine over traditional therapy for the treatment of renal colic in the emergency department, the available evidence indicates that the analgesic effects of intravenous lidocaine may not exceed that of NSAIDS or opiates. Although evidence exists to suggest the efficacy of intravenous lidocaine as an alternative treatment modality for acute renal colic, additional study is needed to clarify its role as compared to other traditional treatment modalities.

## Differences between protocol and review

Due to significant methodological heterogeneity between studies, the planned meta-analysis was not performed.
